# Development and evaluation of the screening performance of a low-cost high-risk screening strategy for breast cancer

**DOI:** 10.20892/j.issn.2095-3941.2020.0758

**Published:** 2021-09-28

**Authors:** Yubei Huang, Huan Wang, Zhangyan Lyu, Hongji Dai, Peifang Liu, Ying Zhu, Fengju Song, Kexin Chen

**Affiliations:** 1 Department of Cancer Epidemiology and Biostatistics, Tianjin Medical University Cancer Institute and Hospital, National Clinical Research Center for Cancer, Key Laboratory of Cancer Prevention and Therapy of Tianjin, Tianjin’s Clinical Research Center for Cancer, Key Laboratory of Cancer Molecular Epidemiology of Tianjin, Key Laboratory of Breast Cancer Prevention and Therapy, Ministry of Education, Tianjin 300060, China; 2 Department of Breast Imaging, Tianjin Medical University Cancer Institute and Hospital, National Clinical Research Center for Cancer, Key Laboratory of Cancer Prevention and Therapy of Tianjin, Tianjin’s Clinical Research Center for Cancer, Key Laboratory of Cancer Molecular Epidemiology of Tianjin, Key Laboratory of Breast Cancer Prevention and Therapy, Ministry of Education, Tianjin 300060, China

**Keywords:** Cancer screening, breast cancer, high risk, mammography, ultrasonography

## Abstract

**Objective:**

To develop and evaluate the screening performance of a low-cost high-risk screening strategy for breast cancer in low resource areas.

**Methods:**

Based on the Multi-modality Independent Screening Trial, 6 questionnaire-based risk factors of breast cancer (age at menarche, age at menopause, age at first live birth, oral contraceptive, obesity, family history of breast cancer) were used to determine the women with high risk of breast cancer. The screening performance of clinical breast examination (CBE), breast ultrasonography (BUS), and mammography (MAM) were calculated and compared to determine the optimal screening method for these high risk women.

**Results:**

A total of 94 breast cancers were detected among 31,720 asymptomatic Chinese women aged 45–65 years. Due to significantly higher detection rates (DRs) and suitable coverage of the population, high risk women were defined as those with any of 6 risk factors. Among high risk women, the DR for BUS [3.09/1,000 (33/10,694)] was similar to that for MAM [3.18/1,000 (34/10,696)], while it was significantly higher than that for the CBE [1.73/1,000 (19/10,959), *P* = 0.002]. Compared with MAM, BUS showed significantly higher specificity [98.64% (10,501/10,646) *vs.* 98.06% (10,443/10,650), *P* = 0.001], but no significant differences in sensitivity [68.75% (33/48) *vs.* 73.91% (34/46)], positive prediction values [18.54% (33/178) *vs.* 14.11% (34/241)], and negative prediction values [99.86% (10,501/10,516) *vs.* 99.89% (10,443/10,455)]. Further analyses showed no significant difference in the percentages of early stage breast cancer [53.57% (15/28) *vs.* 50.00% (15/30)], lymph node involvement [22.73% (5/22) *vs.* 28.00% (7/25)], and tumor size ≥ 2 cm [37.04% (10/27) *vs.* 29.03% (9/31)] between BUS and MAM. Subgroup analyses stratified by breast densities or age at enrollment showed similar results.

**Conclusions:**

The low-cost high-risk screening strategy based on 6 questionnaire-based risk factors was an easy-to-use method to identify women with high risk of breast cancer. Moreover, BUS and MAM had comparable screening performances among high risk women.

## Introduction

Breast cancer is the most common cancer in the world, with an estimated 2.3 million new cases diagnosed in 2020 (11.7% of all cancers)^1,2^. It is the most common cancer in women, both in more and less developed regions, with slightly more cases in less developed than in more developed regions^1,3^. Moreover, breast cancer mortality rates have been stable or decreasing since around 1990 in North America and high resource European countries, while the mortality rates have shown no obvious decrease, but rather potential increases in low resource countries, such as China, due to unique physiological and reproductive characteristics, dramatic lifestyle changes associated with westernization, and the delayed introduction of effective breast cancer screening programs^4–11^.

Several studies suggested that general population-based mammography (MAM) screening could reduce breast cancer mortality^12,13^. Due to limited resources and the relatively low incidence of breast cancer^14^, it is not appropriate to conduct general population-based screening in low resource areas, such as China. On the contrary, high risk population-based screening may be a more suitable choice for China. According to guideline recommendations from the American Cancer Society, the U.S. Preventive Services Task Force, National Comprehensive Cancer Network (NCCN), and the American College of Radiology (ACR)^15–18^, women with high risk of breast cancer were usually defined as women with known underlying genetic mutations (such as a *BRCA1* or *BRCA2* gene mutation or other familial breast cancer syndromes), extremely dense breasts on MAM, or other complex risk prediction models. For residents in low resource areas, it would be cost-prohibitive to conduct tests of BRCA gene mutations, tumor biomarkers, and other blood markers before screening, while it would be also impractical to determine complex cancer risk predictions before screening. Development of a low cost, easy-to-use, and relatively scientific high risk screening strategy would be the preferred choice for low resource areas, such as China.

Until now, there has been no consensus on which breast cancer screening method is more suitable for Asian women, when compared to European or American women^10^. Due to several objective reasons, such as the inaccessibility of MAM equipment, the lack of insurance coverage, and the lack of professional screening technicians, it is impractical to conduct MAM-based breast cancer screening in low resource areas^14^. In China, MAM also seems to be less attractive compared to breast ultrasonography (BUS) in low resource areas due to potential radiation risks, over diagnosis, and less sensitivity of suspicious breast cancers in women with small and dense breasts^19–22^. More recently, several studies reported that BUS showed comparable screening performances compared to MAM in high risk women^19,22^. However, as mentioned above, high risk women were mainly defined by complex models or high cost tests. Few studies have determined whether the screening performances of BUS are also comparable to MAM among high risk women as defined by low cost methods.

Based on the Multi-modality Independent Screening Trial (MIST) of breast cancer in China, we therefore aimed to develop a low-risk, easy-to-use, and relatively scientific method for identifying women at high risk of breast cancer, and then to determine the optimal screening methods for these women in low resource areas.

## Materials and methods

### Introduction of MIST

MIST aimed to evaluate and compare the screening performances of clinical breast examinations (CBEs), BUS, and MAM among Chinese women aged 45~65 years, and to identify a suitable breast cancer screening strategy for targeted women. Detailed information of MIST has been described in our previously reports^4,10,23^. Briefly, a total of 33,234 asymptomatic women who were aged 45~65 years and lived in local communities for more than 3 years were recruited from 5 areas in China (Tianjin, Beijing, Nanchang, Shenyang, and Feicheng) between July 2008 and December 2010. After obtaining informed consent, all participants received a face-to-face questionnaire-based interview conducted by local investigators to collect information on demographic characteristics (such as age, gender, race, marital status, education, income, and insurance), family history of cancer, history of benign breast disease, and factors associated with breast cancer risk, including age of the first menarche, menopausal status, age at menopause, abortion, giving births, breast feeding, oral contraceptive use, and hormone replacement therapy. Body weight (kg) and height (m) were measured by trained investigators, and the body mass index was calculated as the weight in kg divided by the square of height in meters (kg/m^2^). This study was reviewed and approved by the institutional review board of Tianjin Medical University Cancer Institute and Hospital (TMUCIH)(Approval No. bc2018015).

### Screening methods

After a questionnaire-based interview, all participants received CBE, BUS, and MAM. The physicians performed these 3 examinations blindly and separately. All physicians had at least 5 years of work experience regarding the corresponding examinations. All examinations followed unified technical protocols developed by the expert committees of MIST. Bilateral MAM was conducted with a full-field digital mammography system. Bilateral BUS was performed with color Doppler and high resolution transducers with a maximum frequency of at least 10 MHz.

The results of these 3 examinations, including breast mass, calcification, breast density, and other imaging characteristics, were recorded in predesigned case report forms. The results of CBE and BUS were assessed categorically as follows: 1) normal; 2) abnormal benign; 3) suspicious malignancy; and 4) highly suggestive of a malignancy. The results of MAM were assessed according to the Breast Imaging Reporting and Data System of the American College of Radiology (ACR): 0) additional imaging needed; 1) negative; 2) benign finding; 3) probably benign finding; 4) suspicious malignancy; and 5) highly suggestive of a malignancy. All assessments of MAM and BUS were double-checked at primary screening sites. Disagreement between 2 MAM physicians was reassessed by another more experienced MAM physician, while disagreement between 2 BUS physicians was also reassessed by another more experienced BUS physician. Moreover, a subsample of MAM images was sent to TMUCIH for concordance analyses. Detailed information of these methods can be found in our previously published papers^10,23^.

### Follow-up and reference standard

Patients with suspicious malignancies and highly suggestive of breast cancers from any of the abovementioned 3 examinations were immediately recommended for pathological examination. The diagnosis of breast cancer was based on combinations of pathological examinations, clinical diagnoses, or follow-ups within 1 year after the initial screenings. For all breast cancers confirmed by the reference standards, clinical data on tumor characteristics (tumor stage, lymph node involvement, and tumor size) were obtained from pathological reports. The tumor stage was defined according to the American Joint Committee on Cancer Tumor Node Metastasis staging system.

### Development of a simple high risk screening strategy

Several risk prediction models have been previously developed to identify the potential high risk population of breast cancers^24–29^. However, as mentioned above, high risk women were mainly defined by complex models, and the utility of these complex breast cancer risk assessment models was very low. Lack of familiarity was the most cited barrier in the use of these complex risk prediction models^30,31^. Instead of developing a complex risk prediction model, the number of known risk factors of breast cancer was therefore used to identify potential high risk women in this study.

**Figure 1:**
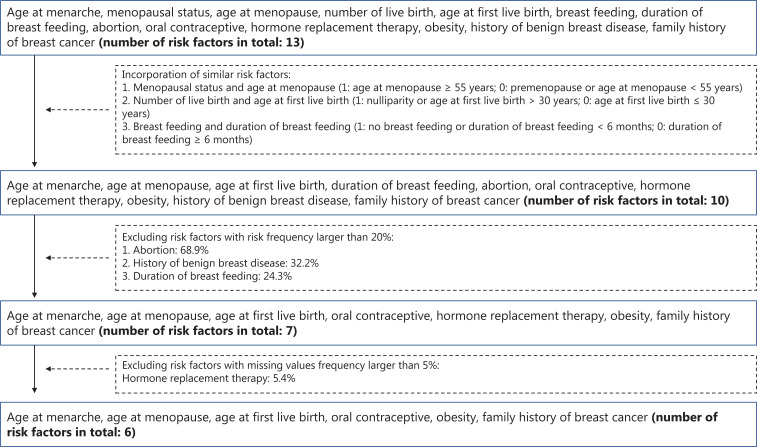
Flowchart of selection factors to determine potential high risk women.

According to our previous studies and other comprehensive studies on the risk factors of breast cancer among Chinese women^6–11,28,32–34^, a total of 13 factors (age at menarche, menopausal status, age at menopause, number of live births, age at first live birth, breast feeding, duration of breast feeding, abortion, oral contraceptive, hormone replacement therapy, obesity, history of benign breast disease, and family history of breast cancer) were initially selected. After excluding correlated factors, factors with risk frequencies ≥ 20% (to avoid labeling too many women as “high-risk groups”)^19^, and factors with missing values ≥ 5%, 6 target factors [age at menarche (AGEMENA), age at menopause (AGEMENO), age at first live birth (AGEFLB), oral contraceptive (OC), obesity, and a family history of breast cancer (FHBC)] were finally selected (**Figure 1**). Among 33,234 participants, after excluding 1,514 (4.6%) women with missing values of the 6 abovementioned target factors, 31,720 women were included in the final analyses.

### Statistical analysis

Pearson’s chi-squared tests and Fisher’s exact tests were used to determine the differences in breast cancer detection for different high-risk screening strategies with different numbers of breast cancer risk factors. Both the breast cancer detection and population coverage of different high-risk screening strategies were used to determine the optimal strategy for defining high risk populations of breast cancer. Among high risk women, Pearson’s chi-squared tests and Fisher’s exact tests were further used to compare breast cancer detection rates for 3 different screening methods (CBE, BUS, and MAM). Pearson’s chi-squared and McNemar’s chi-squared tests with continuity correction were used to compare the screening accuracy [including sensitivity, specificity, positive/negative predictive value (PPV/NPV)] and tumor characteristics (tumor stage, lymph-node involvement, and tumor size) of 3 screening modalities (CBE, BUS, and MAM) among high risk women. Subgroup analyses were further conducted to compare the sensitivities of BUS *vs.* MAM among high risk women by age at enrollment or by breast density.

All the analyses were conducted with R software, version 3.6.2 (The R Foundation for Statistical Computing, Vienna, Austria) and SPSS statistical software for Windows, version 24 (SPSS, Chicago, IL, USA). Two-sided *P* < 0.05 was considered statistically significant.

## Results

### Determination of a high risk screening strategy for Chinese women

**Figure 2:**
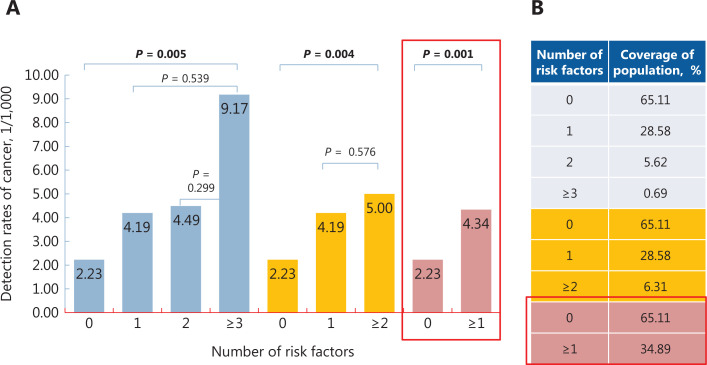
Detection of breast cancer (A) and the coverage of the population (B) according to different numbers of risk factors.

A total of 94 breast cancers were detected among 31,720 asymptomatic Chinese women aged 45–65 years. The characteristics of the population and their corresponding detection of breast cancer are shown in **Supplementary Table S1**. As shown in **Figure 2A and Supplementary Table S2**, the detections were 2.23/1,000 (46/20,654), 4.19/1,000 (38/9,065), 4.49/1,000 (8/1,783), and 9.17/1,000 (2/218) for women with 0, 1, 2, and ≥ 3 risk factors, respectively (*P* = 0.011).

Among women with ≥ 2 risk factors, the detection [5.00/1,000 (10/2,001)] was significantly higher than that among women with < 2 risk factors (*P* = 0.005). However, the percentage of the included population to the whole population was only 6.31%, while a total of 89.36% (84/94) cancers were missed (**Figure 2B and Supplementary Table S2**). We therefore redefined the high risk women as those with any 1 of the 6 listed risk factors. The detection among high risk women [4.34/1,000 (48/11,066)] was still significantly higher than that among low risk women [2.23/1,000 (46/20,654)] (*P* = 0.001) (**Figure 2A**), with 34.89% for the high risk population relative to the entire population (**Figure 2B**). Due to the high detection of breast cancer and relatively suitable coverage of the whole population, the high risk women in this study was finally defined as women who had any of the 6 questionnaire-based risk factors.

### Comparisons of screening performances between different modalities among high risk women

**Figure 3:**
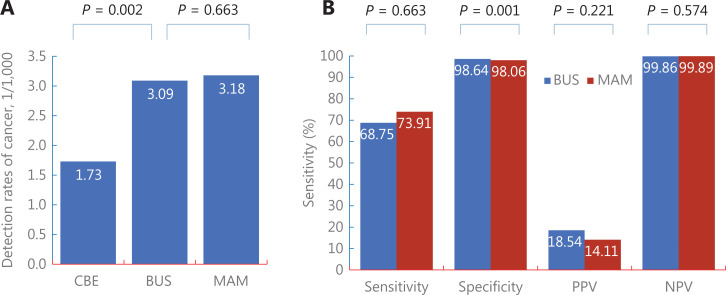
Comparison of detection (A) and screening accuracy (B) between 3 screening modalities among high risk Chinese women. CBE, clinical breast examination; BUS, breast ultrasonography; MAM, mammography; PPV/NPV, positive/negative predictive value, DR, detection rate.

As shown in **Figure 3A and Supplementary Table S3**, among high risk women, the detection for BUS [3.09/1,000 (33/10,694)] was similar with that for MAM [3.18/1,000 (34/10,696), *P* = 0.663], while it was significantly higher for CBE [1.73/1,000 (19/10,959), *P* = 0.002]. Therefore, in order to determine the optimal screening method for high risk Chinese women, the following comparisons of screening performances were conducted between MAM and BUS.

Among screening-detected cancers (**Figure 3B and Supplementary Table S3**), compared with MAM, BUS showed significantly higher specificities [98.64% (10,501/10,646) *vs.* 98.06% (10,443/10,650), *P* = 0.001], but no significant differences in sensitivity [68.75% (33/48) *vs.* 73.91% (34/46), *P* = 0.580], PPV [18.54% (33/178) *vs.* 14.11% (34/241), *P* = 0.221], and NPV [99.86% (10,501/10,516) *vs.* 99.89% (10,443/10,455), *P* = 0.574].

**Figure 4:**
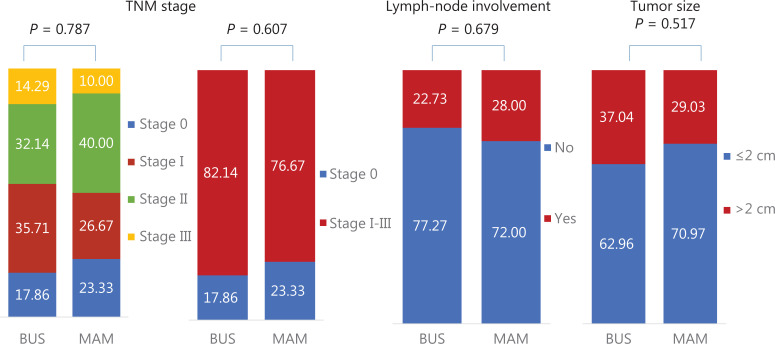
Comparison of tumor characteristics between ultrasonography and mammography among high risk Chinese women.

Further analyses also showed no significant differences in the characteristics of screening-detected cancers between BUS and MAM, including the percentages of early stage (stage 0 + I) breast cancer: [53.57% (15/28) *vs.* 50.00% (15/30), *P* = 0.993], lymph-node involvements [22.73% (5/22) *vs.* 28.00% (7/25), *P* = 0.679], and tumor size ≥ 2 cm [37.04% (10/27) *vs.* 29.03% (9/31), *P* = 0.517] for BUS and MAM, respectively (**Figure 4 and Supplementary Table S4**).

### Subgroup analysis

**Figure 5:**
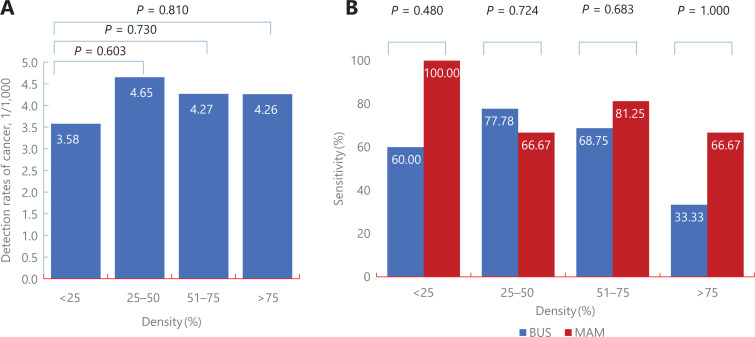
Subgroup analyses of comparisons of screening performances between ultrasonography and mammography according to breast density among high risk Chinese women.

Among high risk women, there was no significant difference in the detection between women with different breast densities, with detections of 3.58/1,000 (5/1,398), 4.65/1,000 (18/3,869), 4.27/1,000 (16/3,749), and 4.26 (3/704) for women with breast densities of 0 < 25% (using control group), 25%–50% (*P* = 0.794), 51%–75% (*P* = 0.979), and > 75% (*P* = 0.059), respectively. Further analyses showed no significant difference of sensitivities between MAM and BUS across 4 subgroups of breast densities, with the sensitivities being 100% (5/5) *vs.* 60% (3/5) for women with breast densities of 0 < 25% (*P* = 0.444), 66.67% (12/18) *vs.* 77.78% (14/18) for women with breast densities of 25%–50% (*P* = 0.457), 81.25% (13/16) *vs.* 68.75% (11/16) for women with breast densities of 51%–75% (*P* = 0.414), and 66.67% (2/3) *vs.* 33.33% (1/3) for women with breast densities of > 75% (*P* = 1.000) (**Figure 5 and Supplementary Table S5**).

**Figure 6:**
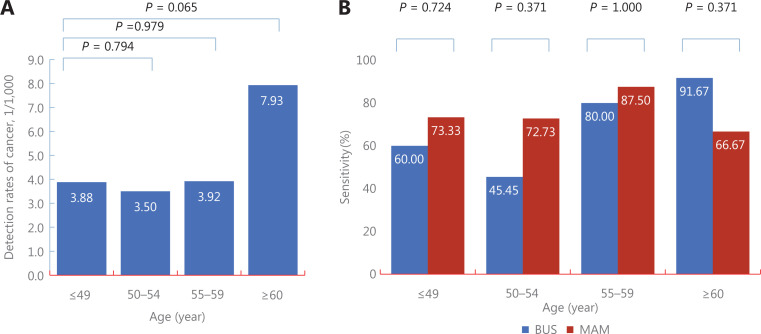
Subgroup analyses of comparisons of screening performances between ultrasonography and mammography according to age at enrollment among high risk Chinese women.

Among high risk women, there was also no significant difference in detection between women with ages at enrollment, with detection of 3.88/1,000 (15/3,863), 3.50/1,000 (11/3,142), 3.92/1,000 (10/2,548), and 7.93 (12/1,513) for women with ages at enrollment ≤ 49 years (Control group), 50–54 years (*P* = 0.794), 55–59 years (*P* = 0.979), and ≥ 60 years (*P* = 0.059), respectively. Further analyses also showed no significant difference of sensitivities between MAM and BUS across 4 subgroups of ages at enrollment, with sensitivities of 73.33% (11/15) *vs.* 60.00% (9/15) for women aged ≤ 49 years (*P* = 0.439), 72.73% (8/11) *vs.* 45.45% (5/11) for women aged 50–54 years (*P* = 0.193), 87.50% (7/8) *vs.* 80.00% (8/10) for women aged 55–59 years (*P* = 1.000), and 66.67% (8/12) *vs.* 91.67% (11/12) for women aged ≥ 60 years (*P* = 0.317) (**Figure 6 and Supplementary Table S5**).

## Discussion

Many low and middle income developing countries cannot afford general population-based MAM screening for breast cancer, including China. Even in developed regions of China, such as Hong Kong, MAM screening is also cost-prohibitive^14,35^. Identifying an affordable screening strategy is an urgent need in response to the increasing burden of breast cancer in China^5,36,37^. In this study, to facilitate the management of high risk women and then conduct high risk population screening in primary communities in China, we first proposed an easy-to-use method for defining a high risk population. In the high risk population, we found that the performance of BUS was comparable to MAM in addition to showing a higher rate of specificity for BUS. These results were similar to previous studies^19,20^.

Since the first risk prediction model, namely the Gail model, was developed in 1989^38^, several risk prediction models for breast cancer have been developed^25,26,39–44^, and numerous studies have been conducted to validate the performance of these risk models^45–49^. However, due to large population heterogeneities and different variables used in these models, the performance of these models has varied across different studies^29,44,45^. Although several modified models had been developed for specific populations, such as Chinese women^28,50^, the utility of these breast cancer risk assessment models by primary care physicians was very low. As reported in the Corbelli et al.^31^ study, only 40% of primary care providers reported having used the Gail model. Even after implementation of an educational program, only 3.8% of eligible patients received breast cancer risk assessment provided by primary care physicians^30^. Lack of familiarity was the most cited barrier in the use of these complex risk prediction models. Simplification of these models would therefore be the first choice to promote the utility of a breast cancer risk assessment tool by primary care physicians.

In addition to the easy-to-use high risk definition method, the more important implication of our study was the similar screening performances of BUS and MAM among high risk Chinese women, which were also reported in the ACRIN 6666 and the study by Shen et al.^19,20^. In both studies, BUS detected significantly more invasive and more negative node cancers than MAM. We also observed similar but not significant results, which was probably caused by the small sample size (**Figure 4**). Moreover, based on the International Agency for Research on Cancer (IARC) Working Group and systematic reviews on the benefits and harms of breast cancer screening^12,13,50^, methods which could detect more invasive but node-negative cancers would be preferred in the future to avoid potential over diagnosis, especially in regions or countries with limited social resources. Based on the results of our study and previous studies, BUS is more preferred than MAM for high risk Chinese women according to the abovementioned prerequisites. However, more studies are needed in the future to support BUS screening.

Several reasons could explain the similar performances of BUS *vs.* MAM among high risk Chinese women. First, Chinese women tend to have smaller and more dense breasts than Americans in a younger age group^10^, which has been reported to reduce the sensitivity of MAM^51^. Second, the peak age at diagnosis of breast cancer among Chinese women was nearly 10–20 years younger than that in American women^14^, and MAM was also less effective in younger women compared with older women^52^. Third, BUS had more potential to detect node-negative and invasive breast cancers than MAM in young women or women with dense breasts^19,20^. We also observed similar results in the subgroup analyses. However, we did not find significant advantages for BUS *vs.* MAM among Chinese young women or women with dense breasts. Small sample size was the probable reason. Moreover, we were not the first to report these results. In the ACRIN 6666 study, although BUS was more likely to detect node-negative and invasive cancers than MAM, there was also no significant difference in the screening sensitivities of BUS *vs.* MAM among young women or women with dense breasts. More studies in the future are therefore needed to confirm the reasons for the similar performances of BUS *vs.* MAM among high risk women.

In addition to the aforementioned findings, this study also had certain limitations. First, although we proposed an easy-to-use method with 6 risk factors to identify potential high risk women, missed diagnoses were inevitable, as shown in **Supplementary Table S2**, a total of 46 cancers were reported among 20,654 participants with 0 risk factors. In the future, these individuals should be initially screened by combining traditional cancer risk assessment, genetic risk assessment, tumor markers, or other routine indicators (such as blood lipids) to avoid potential missed diagnoses. In addition, as shown in **Supplementary Table S1**, significant differences in the detection of breast cancer in different subgroups were only found for AGEMENO and FHBC, but not for the other 4 variables. This limitation was probably caused by too few cases and too many healthy participants in the screening studies, where it is often impossible to confirm all significant associations observed in case-control studies. Second, to develop a feasible high risk screening strategy for breast cancer in low resource areas, it is necessary to conduct health economics evaluations based on the effectiveness and costs of screening. However, this study did not collect necessary cost information required in cost evaluations, such as the time cost for participating in screening, the cost of further diagnostic examinations and treatment for screening-detected cancer patients, and the comparable costs of treatments for patients diagnosed in clinical visits rather than by screening during the same period. The current results were therefore not available for health economics evaluations. More studies are needed in the future to explore the cost-effectiveness ratio of this high risk screening strategy. Third, due to the lack of long-term follow-ups, it was impossible to evaluate the impact of the high risk screening strategy proposed in this study on breast cancer mortality. However, as reported in the latest cancer registry report in China, the crude breast cancer incidence was 45.29 per 100,000^53^, which was significantly lower than the detection of breast cancer (3.09/1,000) in our study (**Figure 3**). The percentage of early stage breast cancer in our study (53.57%, **Figure 4**) was also significantly higher than that among clinically-diagnosed breast cancer (20.1%)^54^. Based on the high detection and the high percentage of early stage breast cancers, we can expect that the high risk screening strategy proposed in this study will lead to a reduced breast cancer mortality in the future.

## Conclusions

In summary, we have proposed an easy-to-use high-risk screening strategy for breast cancer. BUS showed similar screening performances with MAM in the cancer detection, accuracy, and tumor characteristics among Chinese high risk women. Although further validation studies are needed to confirm these results, this study suggested that BUS is a potentially preferable method, when compared to MAM for high risk Chinese women, particularly in regions where there is a lack of MAM equipment but with more BUS systems.
