# Supplementary material for: Development and evaluation of the screening performance of a low-cost high-risk screening strategy for breast cancer
Source: Cancer Biol Med. 2021 Sep 28;19(9):1375–84. doi: 10.20892/j.issn.2095-3941.2020.0758 (PMC9500221; doi:10.20892/j.issn.2095-3941.2020.0758)
Supplement: Supplementary file 1 [file cbm-19-1375-s001.pdf]

Supplementary materials

Table S1 Characteristics of the population and their corresponding detections of breast cancer

Variable	Participants (*n* = 31,720)	Non-case (*n* = 31,626)	Case (*n* = 94)	Detection rate, 1/1,000	*P*
AGEMENA					
No	28,321	28,238	83	2.93	0.757
Yes	3,399	3,388	11	3.24	
AGEMENO					
No	30,860	30,774	86	2.79	0.001
Yes	860	852	8	9.30	
AGEFLB					
No	29,440	29,356	84	2.85	0.195
Yes	2,280	2,270	10	4.39	
OC					
No	28,500	28,420	80	2.81	0.127
Yes	3,220	3,206	14	4.35	
OBESITY					
No	28,980	28,897	83	2.86	0.290
Yes	2,740	2,729	11	4.01	
FHBC					
No	30,921	30,833	88	2.85	0.017
Yes	799	793	6	7.51	

AGEMENA, age at menarche; AGEMENO, age at menopause; AGEFLB, age at first live birth; OC, oral contraceptive; OBESITY, obesity, FHBC, family history of breast cancer.

Table S2 Detection of breast cancer according to the different numbers of risk factors

Number of risk factors	Case (*n* = 94)	Participants (*n* = 31,720)	Detection rate, 1/1,000	Coverage of participants, %	*P*
0	46	20,654	2.23	65.11	0.011
1	38	9,065	4.19	28.58	
2	8	1,783	4.49	5.62	
≥ 3	2	218	9.17	0.69	
Reclassification method 1					
0	46	20,654	2.23	65.11	0.005
1	38	9,065	4.19	28.58	
≥ 2	10	2,001	5.00	6.31	
Reclassification method 2					
0	46	20,654	2.23	65.11	0.001
≥ 1	48	11,066	4.34	34.89	

Table S3 Comparison of detection and screening accuracy between 3 screening modalities among high risk Chinese women

Method	Result	Non-case	Case	Participants	Sensitivity, %	Specificity, %	PPV, %	NPV, %	DR, 1/1,000
CBE	Negative	10,843	29	10,872	39.58	99.38	21.84	99.73	1.73
	Positive	68	19	87					
	Total	10,911	48	10,959					
BUS	Negative	10,501	15	10,516	68.75	98.64	18.54	99.86	3.09
	Positive	145	33	178					
	Total	10,646	48	10,694					
MAM	Negative	10,443	12	10,455	73.91	98.06	14.11	99.89	3.18
	Positive	207	34	241					
	Total	10,650	46	10,696					

CBE, clinical breast examination; BUS, breast ultrasonography; MAM, mammography; PPV/NPV, positive/negative predictive value, DR, detection rate.

Table S4 Comparison of tumor characteristics between ultrasonography and mammography among high risk Chinese women

Tumor characteristics	BUS (n = 33)		MAM (n = 34)		P
	n	%	n	%	
TNM stage					
Stage 0	5	17.86	7	23.33	0.787
Stage I	10	35.71	8	26.67	
Stage II	9	32.14	12	40.00	
Stage III	4	14.29	3	10.00	
Stage 0 + I	15	53.57	15	50.00	0.993
Stage II + III	13	46.43	15	50.00	
Lymph node involvement					
No	17	77.27	18	72.00	0.679
Yes	5	22.73	7	28.00	
Tumor size					
≤ 2 cm	17	62.96	22	70.97	0.517
> 2 cm	10	37.04	9	29.03	

Table S5 Subgroup analyses of comparisons of screening performances between ultrasonography and mammography according to breast density and age at enrollment among high risk Chinese women

Screen characteristic No. cancers/ No. women		Screens with cancer		BUS		MAM		Difference in US *vs.* mammography	
		Detection rate, 1/1,000	No. detected/ No. cancers	Sensitivity, %	No. detected/ No. cancers	Sensitivity, %	Estimate, %	*P*	
Density, %	< 25	5/1,398	3.58	3/5	60.00	5/5	100.00	−20.00	0.444
	25–50	18/3,869	4.65	14/18	77.78	12/18	66.67	11.11	0.457
	51–75	16/3,749	4.27	11/16	68.75	13/16	81.25	−12.50	0.414
	> 75	3/704	4.26	1/3	33.33	2/3	66.67	−33.33	1.000
	P trend	0.886		0.467		0.666			
Age at enrollment, year	≤ 49	15/3,863	3.88	9/15	60.00	11/15	73.33	−13.33	0.439
	50–54	11/3,142	3.50	5/11	45.45	8/11	72.73	−27.27	0.193
	55–59	10/2,548	3.92	8/10	80.00	7/8	87.50	−7.50	1.000
	≥ 60	12/1,513	7.93	11/12	91.67	8/12	66.67	25.00	0.317
	P trend	0.111		0.036		0.874			
